# Aging of the Microenvironment Influences Clonality in Hematopoiesis

**DOI:** 10.1371/journal.pone.0042080

**Published:** 2012-08-06

**Authors:** Virag Vas, Katharina Senger, Karin Dörr, Anja Niebel, Hartmut Geiger

**Affiliations:** 1 Department of Dermatology and Allergic Diseases, University of Ulm, Ulm, Germany; 2 Division of Experimental Hematology and Cancer Biology, Cincinnati Children’s Hospital Medical Center, University of Cincinnati, Cincinnati, Ohio, United States of America; RWTH Aachen University Medical School, Germany

## Abstract

The mechanisms of the age-associated exponential increase in the incidence of leukemia are not known in detail. Leukemia as well as aging are initiated and regulated in multi-factorial fashion by cell-intrinsic and extrinsic factors. The role of aging of the microenvironment for leukemia initiation/progression has not been investigated in great detail so far. Clonality in hematopoiesis is tightly linked to the initiation of leukemia. Based on a retroviral-insertion mutagenesis approach to generate primitive hematopoietic cells with an intrinsic potential for clonal expansion, we determined clonality of transduced hematopoietic progenitor cells (HPCs) exposed to a young or aged microenvironment *in vivo.* While HPCs displayed primarily oligo-clonality within a young microenvironment, aged animals transplanted with identical pool of cells displayed reduced clonality within transduced HPCs. Our data show that an aged niche exerts a distinct selection pressure on dominant HPC-clones thus facilitating the transition to mono-clonality, which might be one underlying cause for the increased age-associated incidence of leukemia.

## Introduction

Aging is a major risk factor for leukemia. The mechanisms of the age-associated exponential increase in the incidence of leukemia are not known in detail, but it is widely accepted that the combination of leukemia initiating cell-intrinsic as well as extrinsic mechanisms drive this process. [1]–[5] While the contribution of hematopoietic cell intrinsic mechanisms to the increase in leukemia with age have been described [6], [7], whether aging of the bone marrow (BM) microenvironment also influences pre-leukemic states or leukemia has not been studied in great detail so far. [8], [9] The marrow microenvironment is able to influence differentiation [10], drug resistance [11] and proliferation of leukemic cells in diverse mouse leukemia-models [12], [13] while it undergoes multiple changes upon aging as demonstrated by decreased bone-remodeling [14], enhanced adipogenesis and changes in ECM components [15], [16].

Clonality is tightly linked to initiation of leukemia. [17] We consequently tested whether the age of the microenvironment influences *in vivo* clonality among dominant hematopoietic progenitor cells obtained via retroviral insertional mutagenesis. In summary our data support the novel concept that an aged niche exerts a distinct selection pressure on dominant HPC-clones that eases the transition to monoclonality, which might be one underlying cause for the increased age-associated incidence of leukemia.

## Results and Discussion

To test whether an aged microenvironment influences *in vivo* clonality, BM depleted of mature cells (lin-) were transduced with the SF91/IRES-eGFP replication incompetent retrovirus, a set-up similar to insertional mutagenesis screens. The SF91 virus confers a high potential to generate *in vivo* dominant HPSCs by activation of proto-oncogenes in the neighborhood of the retroviral integration site (RIS), while the frequency of malignant transformation remains low [18], [19].

**Figure 1:**
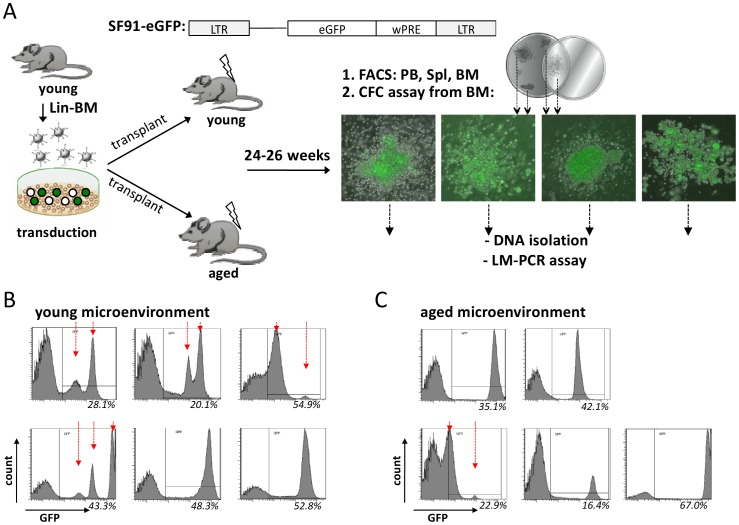
The clonal composition of the hematopoiesis is different in old compared to young microenvironment. (A) Schematic representation of the gammaretroviral SF91/IRES-eGFP vector used for insertional mutagenesis and the experimental setup (LTR: long terminal repeat with strong enhancer element, wPRE: woodchuck hepatitis virus posttranscriptional regulatory element). Lineage depleted (lin-) BM cells from young C57BL/6 mice were pre-stimulated with a cytokine cocktail and transduced. Cells were subsequently transplanted in equal amounts into young and aged recipient mice. (The graft contained 51.6%, 30.7%, 32.4% GFP+ cells in the 3 transduction/transplantation.) 24–26 week post-transplantation recipients were sacrificed and PB, spleen and BM analyzed by flow cytometry for lineage differentiation markers as well as the number of primitive L-S+K+ cells. At the same time CFC-assays on BM cells were performed and DNA from GFP+ colonies (representative pictures) isolated for LM-PCR. GFP expression among BM cells of young **(B)** and aged **(C)** recipient mice. (young mice n = 6, aged mice n = 5, from 3 independent experiments).

Pools of cells from the same transduction procedure were subsequently transplanted into young (2 month) and aged (18–19 months) recipient mice and GFP chimerism in peripheral blood was monitored by flow cytometry to follow the fate of the transduced cell population *in vivo*. 24–26 weeks post transplantation, when hematopoiesis is driven by the transplanted hematopoietic stem cells, GFP+ (transduced) cells in PB, spleen and BM were analyzed in detail (Figure 1A). This experimental set-up combined two goals: (1) The determination of clonality was possible as HSCs were individually marked by the random nature of the genomic site of the retroviral-integration. (2) Integration of this vector elicits retroviral insertional mutagenesis, and the transduced cells are prone to form dominant clones, serving as a model system for the initiation of leukemogenesis.

The BM of the recipient mice showed distinct contribution of GFP+ cells, which might be due to the distinct influence of the viral integration site on HSCs survival as well as the contribution of the niche and microenvironment on HSCs behavior. In order to analyze the effect of a young or aged BM niche on clonality within the transduced and transplanted cells, we focused on animals with more than 15% GFP+ chimerism in bone marrow to perform ligation-mediated PCR (LM-PCR) to determine both integration sites as well as clonality. The 15% threshold for eGFP+ cells was set because at this level of chimerism mutagenesis potentially resulted in a survival advantage of the transduced HSC leading to proliferation/expansion as the first step of pre-leukemic stage, thus fulfilling a basic requirement for our model.

Although the GFP profile of the BM sample has the limitation that it represents differentiated cells and not the progenitor/stem cell population, it can serve as at least an indicator of clonality within differentiated cell populations in hematopoiesis. The GFP expression profile of transduced cells harvested from recipient’s BM revealed that the majority of young recipients presented with multiple GFP peaks (Figure 1B) while transduced cells in the aged microenvironment displayed primarily only one GFP peak (Figure 1C), suggesting differences in clonality within hematopoiesis in an old compared to a young microenvironment.

**Figure 2:**
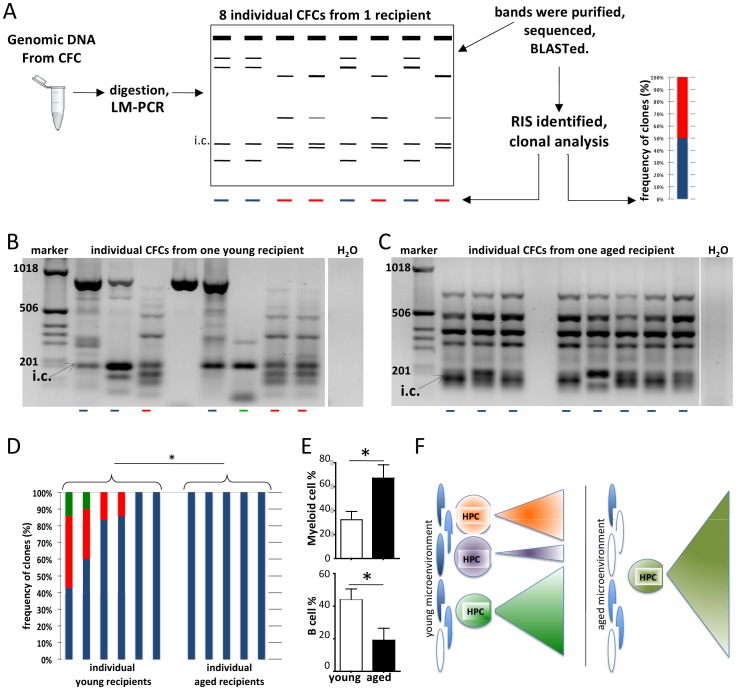
Coexistence of balanced HSC populations is characteristic for young microenvironment whereas an aged microenvironment favors the expansion of single dominant hematopoietic clone. (**A**) Schematic representation of the retroviral insertional mutagenesis screen. DNA was extracted from individual CFCs (average: 7.3±1.8 GFP+CFCs/mouse were isolated) and digested with four-cutter enzymes. After LM-PCR, the recovered bands were isolated and sequenced. The sequences were aligned to the mouse genome with NCBI/BLAST program. The RISs were identified and the closest genes (within a ±100 kb window) were listed. Based on the LM-PCR band-pattern and the identified RISs, clonal analyses were performed. (**B**) Representative agarose gel analysis of LM-PCR performed on methylcellulose colonies isolated from BM of one representative young transplanted mouse. Clonality was identified based on the band pattern and DNA sequence information on individual bands. Distinct colors represent distinct clones. (**C**) Insertion sites recovered by LM-PCR from CFCs of one representative aged recipient mouse. The red arrows depict distinct GFP peaks. i.c.: internal control bands represent PCR products amplified from the viral genome. (**D**) Distribution of clonality within the GFP+ CFC population of young and aged recipient mice determined by the LM-PCR pattern and sequence information. (**E**) Flow cytometric analyses of PB of young and aged recipient mice (same mice were used in clonal analysis and RISs identification). Myeloid cells were identified based on Gr1 and Mac1 expression and B220 was used as B cell marker. (young mice n = 6, aged mice n = 5, from 3 independent experiments), * = p<0.05 (**F**) Proposed model for clonality of hematopoiesis in young and aged microenvironment.

To further investigate clonality in more primitive hematopoietic cells exposed to a young or aged microenvironment, retroviral insertion sites (RISs) from individual GFP+ colony forming cells (CFCs) from BM were amplified by ligation-mediated PCR and genomic location of RISs were determined (Figure 1A). The gel-band pattern of the LM-PCR reaction, in combination with the sequence information from distinct bands were used to determine the frequency of distinct clones in the myeloid progenitor-cell compartment (Figure 2A). As the same pool of transduced graft cells were transplanted into young and aged animals, differences in the two experimental groups in hematopoiesis result primarily from the distinct age of the microenvironment. In young mice, hematopoiesis within the GFP+CFCs remained in the majority oligoclonal as indicated by distinct banding patterns among individual CFC-clones and confirmed by sequencing (Figure 2B,D). GFP+CFCs from individual aged recipients presented under the LM-PCR protocol used without exception a monoclonal pattern (Figure 2C,D). The difference in the clonality pattern among the myeloid stem/progenitor population between young and aged animals was significant (p = 0.035). The LM-PCR method was sensitive enough to demonstrate minor clone contributing down to least 10% of the transduced population (Figure 2D), while the restriction enzyme used for LM-PCR analysis will cover at least 85% of all possible integration sites [20], implying that the observed mono-clonality in CFC colonies might represent in a good number of cases true monoclonality in vivo.

**Table 1:** Insertion sites in CFCs isolated from young and aged recipient mice.

In young microenvironment			In old microenvironment		
Gene ID	Official gene symbol		Gene ID	Official gene symbol	
**Endocytosis GO cluster:**			**Regulation of transcription GO cluster:**		
20403	Itsn2		14013	Mecom, Evi1	
20910	Stxbp1		17863	Myb	
227738	Lrsam1		52915	Zmiz2	
**Genes with miscellaneous function:**			**Genes with miscellaneous function:**		
11534	Adk		12857	Cox4i1	
12014	Bach2/Evi59 *		17909	Myo10	
12540	Cdc42		19252	Dusp1	
12561	Cdh4		22379	Fmnl3	
14297	Fxn		52906	Ahi1	
15403	Hoxa6		54614	Prpf40b	
15404	Hoxa7		67561	Wdr48	
16800	Arhgef2		68581	Tmed 10	
17977	Ncoa1		74498	Gorasp1	
18263	Odc1		75740	Egfem1	
21873	Tjp2		110213	Tmbim6	
23984	Pde10a		217718	Nek9	
53602	Hpcal1		268373	Ppi A	
54169	Myst4		**Genes with unknown function:**		
56459	Sae1		432939	Gm5468	
57915	Tbc1d1		545510	Gm10258	
71801	Plekhf2		666668	Gm8225	
140858	Wdr5		100417604	Gm18709	
227737	Fam129b		100502810	Gm19388	
242093	Rxfp4		100504283	Gm20149	
320150	ZDHHC17				
330474	Zc3h4				
381409	Cdh26				
414084	Tnip3				
435391	Dupd1				
**Genes with unknown function:**					
66931	1700010I14Rik				
67791	6530411M01Rik				
68169	A930038C07Rik				
72628	2700086A05Rik				
76947	2310030N02Rik				
432502	Gm5428				
595140	Gm6044				
665533	Gm13004				
100038651	D130062J21Rik*				
100188958	Gm15824				
100417345	Gm18548				
100417946	Gm18913				
100503024	Gm19512				
100503065	LOC100503065				
100504144	Gm13421				

RISs isolated from CFC mice with a chimerism higher than 15% (also displayed in Figure 2/D, see also Material and Methods S1) are listed. Gene categories were defined by the DAVID gene ontology (GO)^21^, using high classification stringency option by clustering. For Endocytosis GO group, the Fisher Exact test gave a P-value of 4.0E−2 and for Regulation of transcription GO group P-value = 2.9E−1.

* = same RISs were found in 2 different young recipient mice (transplanted with two different pools of transduced cells), all the other RISs were unique within the experiments.

All animals transplanted in this study remained leukemia-free at least up to 6 month post-transplantation as indicated by normal spleen weight (Figure S1) and normal PB white blood cell counts (data not shown) in both age, supporting that the frequency of malignant transformation in response to transduction with SF91 is low, while at the same time generating dominant clones. As the chimerism for GFP positivity in PB, in lineage+ BM (differentiated) cells as well as in the primitive hematopoietic cell compartment (L-S+K+) and the GFP+CFC% in methylcellulose culture were similar in young and aged recipients, and the overall total number of L-S+K+ (GFP+ and GFP-) cells was similar in an aged or young niche (Figure S1), the difference in clonality between young and aged recipients can not be a consequence of an assumed distinct levels of chimerism of transduced/untransduced cells in young and aged recipients. Our data indicates that the same pool of cells, when exposed to an aged instead of a young microenvironment resulted in significantly reduced clonality, ultimately approaching mono-clonality in an aged microenvironment.

The genomic locations of RISs and the nearest neighboring genes within 100 kb of the RIS in the individual CFCs are listed in Table 1 and Table S1. Overall, more RISs were recovered from the oligoclonal young recipient group, because more clones were present in this group than in the monoclonal aged recipient group. As depicted in figure 2A and B, some clones contained more than one RIS and our technique was able to distinguish which RISs belong to individual clones. Genes in close vicinity of the RISs, which are frequently involved in the formation of dominant clones [19] were distinct between CFCs from young and aged recipients, and DAVID gene ontology [21] clustering also revealed distinct GO categories for RISs listed in a young or an aged microenvironment. These data further support that distinct set of cells/CFCs were selected in the two different (young/aged) microenvironments.

Aging in hematopoiesis is associated with a shift in lineage differentiation, in which B-lymphoid output is diminished and myeloid output enhanced, which has been primarily linked to stem cell intrinsic changes with age. [22] Interestingly, and in agreement with recently published data [23], the output of transduced BM cells exposed to aged microenvironment was significantly skewed towards the myeloid lineage with a diminished B-lymphoid contribution (Figure 2E), supporting as another novel concept that also niches, and not only stem cell intrinsic mechanisms are able to induce a lineage shift associated with aging under our dominant clone conditions.

In summary our data support a model in which an aged microenvironment, in contrast to a young one, selects for a few or only a single dominant hematopoietic clone and thus paving the way to mono-clonality (Figure 2F), which might be a contributing factor to the elevated incidence of leukemia in the elderly.

## Materials and Methods

### Retroviral Transduction and Transplantation Conditions

Lineage depleted (lin-) [24] and pre-stimulated young bone marrow (BM) cells were transduced overnight on retronectin-coated (TaKaRa, Otsu, Japan) plates with cell-free supernatants containing SF91/IRES-eGFP retrovirus as described in ref. [18]. See Material and Methods S1. Prior to transplantation, young (2 month) and aged (18–19 month) C57Bl/6 mice were non-lethally irradiated (total-body, 9.5 gray) and approximately 10^6^ cells/recipient were injected. The number of young transplanted mice was 15 and 11 aged mice along with three independent transductions. See in Material and Methods S1.

### Analysis of Recipient Mice

At 24–26 week post-transplant, when hematopoiesis is supported by long-term HSCs, we analyzed all of the 26 recipients for CFC formation among BM cells and also subjected then to FACS analysis. All animals initially transplanted were sacrificed, and PB, BM and spleen were analyzed for lineage markers (CD3, B220, Gr-1, Mac1) and lin−/c-Kit+/Sca1+ cells by flow cytometry as described in ref. [24]. For CFC-assays, 4×10^5^ BM cells from each mice were seeded in methylcellulose culture (M3534, StemCell Technologies) for 9 days, GFP+ colonies were picked and genomic DNA from individual colonies were isolated (Qiagen) for LM-PCR. Only recipients with more than 15% eGFP+ cell frequency (similar frequency in young and aged recipients, see Figure S1 and Figure 1B,C) were further analyzed, as it was anticipated that in these animals the initial formation of dominant clones was successful, while they also gave rise to sufficient numbers of eGFP+CFCs within the numbers plated for further analysis. We then analyzed the eGFP+CFCs from these recipients that provided higher than 15% chimerism by LM-PCR.

### Statistics

T-test was performed to determine the significance of the difference between means of two groups. Values were considered significant when p<0.05.

### Ligation-mediated PCR (LM-PCR) and Retroviral Insertion Sites (RISs) Identification

LM-PCR was performed as described in ref. [25] and Material and Methods S1. PCR fragments from gels were isolated, sequenced (GATC, Konstanz, Germany) and sequences obtained were analyzed with following websites/databases: BLAST(http://blast.ncbi.nlm.nih.gov), Ensembl (http://www.ensembl.org), DAVID [21], RTCGD [26] and iDDb [19]. See in Material and Methods S1.

### Ethics Statement

Animal experiments were carried out in University Ulm accordance with Tierschutzgesetz Paragraph8 Abs.1 and 3, were approved by the “Regierungspräsidium Tübingen” (Az:35/9185.81 protocol number 957). The study was performed in cooperation with the Comprehensive Mouse and Cancer Core in the Division of Experimental Hematology, Cincinnati Children’s Hospital Medical Center in accordance with the approved animal handling protocols (Nr. 9D04039 and 8D10089) provided by the Institutional Animal Care and Use Committee (IACUC) Cincinnati.

## Supporting Information

Figure S1 **Transplanted aged and young mice display normal hematopoiesis. (A)** Spleen weight indicates normal spleen size (up to 120 mg). **(B)** GFP+ cell contribution in PB, differentiated BM (lineage marker positive: lin+) and lin−/c-kit+/Sca1+ (L-S+K+) cell-population 24–26 weeks post-transplant in young and aged recipient mice. The similar GFP levels in the different cell population indicate that transduced cells do not show a differentiation arrest. **(C)** GFP+CFC frequency among the total CFCs in methylcellulose culture of BM cells. **(D)** The size of the primitive cell compartment (LSK cell number) remained in normal range and did not differ from young and aged transplanted mice, which indicates that this cell pool remained under the regulatory control of the niche. (young transplanted mice n = 15, aged transplanted mice n = 11, from a total of 3 independent biological repeats, bars represent the mean ±SEM). Based on the analyzed PB and BM samples, the recipient mice did not show malengraftment.
(TIF)

Table S1 **Detailed list of integration sites isolated from young (A) and aged (B) recipient mice after 24–26 weeks of transplantation.** The genomic locations of RISs and the nearest neighboring genes within 100 kb are listed. The RISs isolated from to the same recipient were grouped together to show that more RISs were recovered from young mice with oligoclonal GFP+population and less insertion sites from the monoclonal GFP+ population of the aged recipients. Figure 2/D was created based on sequencing results in combination with LM-PCR band patterns. The range of integrations per clone was calculated. The RIS/cell ranged from 1 to 4). The listed RISs were compared with two databases (Retroviral Tagged Cancer Gene Database (RTCGD), Insertional Dominance Database (IDDb)) to strengthen the validity of the applied methods. Within the RTCGD, retroviral integration sites associated with candidate cancer genes have been collected (ref. ^22^). The IDDb listed RISs related to dominant clone formation (ref. ^23^). Overall, 27.9% of the recovered RISs from young mice and 27.2% from old were found in the RTCGD, and 3 genes from young recipients and 1 gene from old recipients were found in IDDb. This efficiency to recover already described viral integration sites supports the validity of our approach. * =  insertion sites belong to the dominant clone.
(DOCX)

Materials and Methods S1 **Detailed description of the performed experiments**
(XLSX)
