# Supplementary figures and images for: Aging of the Microenvironment Influences Clonality in Hematopoiesis
Source: PLoS One. 2012 Aug 6;7(8):e42080. doi: 10.1371/journal.pone.0042080 (PMC3412859; doi:10.1371/journal.pone.0042080)

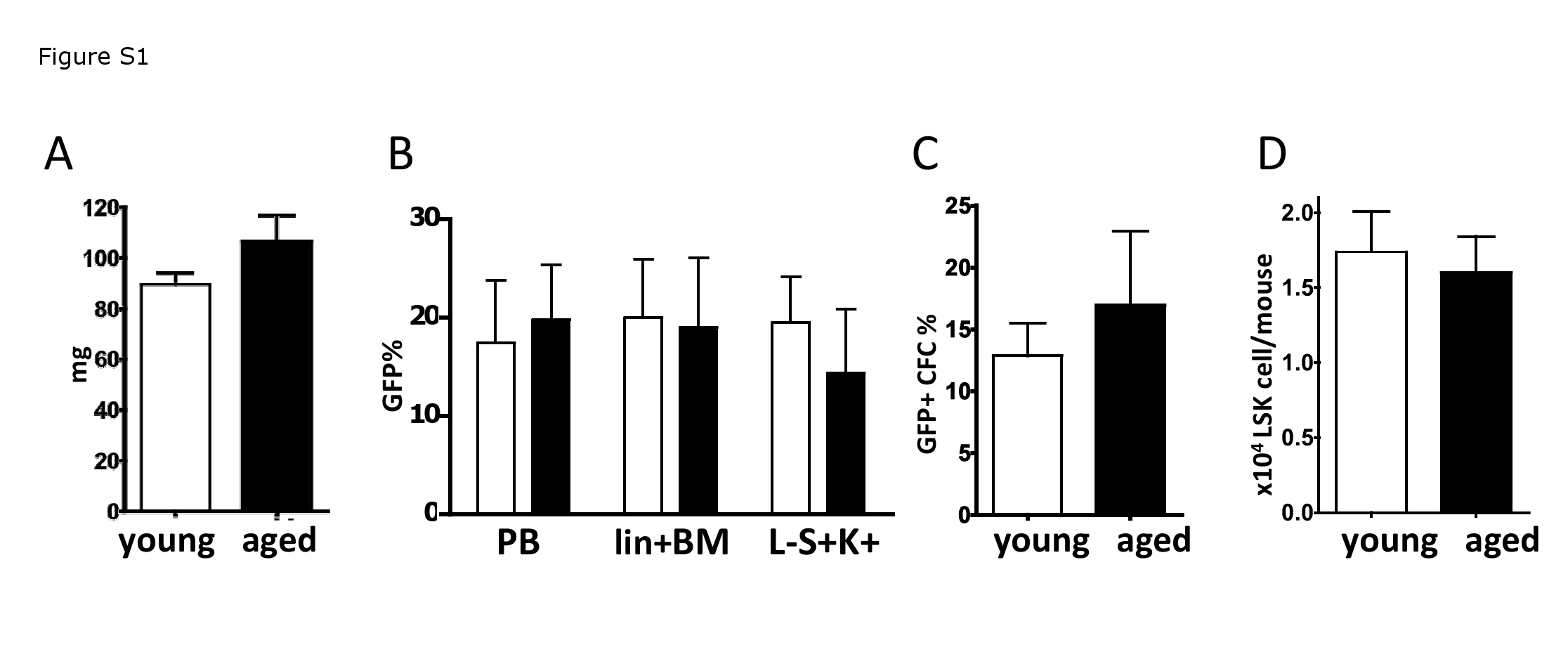

Supplement: Figure S1 — Transplanted aged and young mice display normal hematopoiesis. (A) Spleen weight indicates normal spleen size (up to 120 mg). (B) GFP+ cell contribution in PB, differentiated BM (lineage marker positive: lin+) and lin−/c-kit+/Sca1+ (L-S+K+) cell-population 24–26 weeks post-transplant in young and aged recipient mice. The similar GFP levels in the different cell population indicate that transduced cells do not show a differentiation arrest. (C) GFP+CFC frequency among the total CFCs in methylcellulose culture of BM cells. (D) The size of the primitive cell compartment (LSK cell number) remained in normal range and did not differ from young and aged transplanted mice, which indicates that this cell pool remained under the regulatory control of the niche. (young transplanted mice n = 15, aged transplanted mice n = 11, from a total of 3 independent biological repeats, bars represent the mean ±SEM). Based on the analyzed PB and BM samples, the recipient mice did not show malengraftment. (TIF) [file pone.0042080.s001.tif]
