# Supplementary material for: Aging of the Microenvironment Influences Clonality in Hematopoiesis
Source: PLoS One. 2012 Aug 6;7(8):e42080. doi: 10.1371/journal.pone.0042080 (PMC3412859; doi:10.1371/journal.pone.0042080)
Supplement: Materials and Methods S1 — Detailed description of the performed experiments (XLSX) [file pone.0042080.s003.xlsx]

#### Materials and Methods S1

#### Retroviral Gene Transfer and Bone Marrow Transplantation

BM was flushed from the long bones from 2–3 month old C57BL6 mice and mononuclear cells were isolated by low-density centrifugation (Histopaque 1083, Sigma). The mononuclear cells were incubated with a cocktail of mixture of rat-anti-mouse antibodies recognizing antigens on mouse differentiated cells: CD11b (clone M1/70), B220 (clone RA3-6B2), CD5 (clone 53-7.3) Gr-1 (clone RB6-8C5), Ter119, CD8a (Clone 53-6.7) (all from BD Pharmingen) and depleted with anti-rat-IgG magnetic beads (Invitrogen Dynal). The lin- cells were pre-stimulated in six-well non-tissue culture dishes for 2 days in IMDM medium (Lonza, Verviers, Belgium)/10%FBS (HyClone, Thermo Scientific, South Logan, Utah) containing 50ng/mL mSCF, 100ng/mL Flt-3 ligand, 100ng/mL IL-11, 10ng/mL mIL-3 (Prospec, Tany TechnoGene Ltd., Rehovot, Israel), 1% penicillin/streptomycin, and 2 mM glutamine at a density of 6,5–8×10^6^ cell/well.

The transduction was performed on day 3 in six-well plates on RetroNectin-coated (9.5 µg/cm^2^; TaKaRa, Otsu, Japan) non-tissue culture dishes. Virus preloading was carried out with cell free virus supernatant by centrifugation (40 min., at 4 C, 1300 g). The pre-stimulated lin- cells were seeded on top (1 ml of 8–9×10^5^ prestimulated cells/well) with fresh thawed viral supernatant (2 ml) supplemented with cytokines (mSCF, Flt-3 ligand, IL-11, mIL-3) with an MOI of 1.2±0.25 among three experiments and incubated overnight. By the experimental plan we take care about to minimize the culture period before transplantation, therefore next morning, the transduced cells were collected with cell dissociation buffer (Gibco) and transplanted the graft cells in preconditioned recipient mice. Prior to transplantation the young and old recipient mice were irradiated with 9.5 Gy, and cells were thoroughly mixed and approximately 10^6^ cells/recipient were transplanted into mice by retro-orbital injection.

The graft cells were handled as a bulk culture during the transduction, which allows us to assume, that the same way treated and handled cell population were transplanted into young and aged mice. We concluded that the culture conditions used before transplantation did not have an effect on the clonality of the stem/progenitors in the recipient mice.

#### Ligation Mediated-PCR (LM-PCR)

Genomic DNA isolated from CFC colonies were digested with 5 U of restriction enzyme Tsp509 I or Mse1 (New England BioLabs) in 30ul final reaction-volume for 4 hr at 65°C. After precipitation, biotinylated retroviral-sequence specific primer (A1RV) was used for primer extension reaction with 2.5u Pfu DNA polymerase (Agilent Technologies) (64°C for 30min). To separate the biotinylated sequences, streptavidin coated M-280 Dynabeads (Invitrogen Dynal) were applied for 3hr at RT. Then, the remaining blunt-end of the genomic DNA fragments were ligated with a polylinker cassette with T4 ligase (New England BioLabs).

To amplify the insertion site regions, both first and nested PCR (94°C for 2 min; 94°C for 15 sec, 60°C for 30 sec, 68°C for 1 min for 30 cycles; 68°C for 10min) were performed with Extensor Hi-Fidelity PCR Master Mix (Thermo Fisher Scientific Inc.), linker-specific primers (OCI, OCII), and retroviral-specific primers (A2RV, A3RV), respectively. PCR products were isolated after gel electrophoresis using QIAquick Gel Extraction Kit (QIAGEN, Hilden Germany) and sequenced directly using the primer RAseq. In every procedure as an internal standard to check the reproducibility known standard BM sample was included.

Primers:

A1RV-biot: 5’Biotin-CTGGGGACCATCTGTTCTTGGCCTC-3’

Primer OCI: 5’-GACCCGGGAGATCTGAATTC-3’

Primer OCII: 5’-AGTGGCACAGCAGTTAGG-3’

Primer A2RV: 5’-GCCCTTGATCTGAACTTCTC-3’
Primer A3RV: 5’-CCATGCCTTGCAAAATGGC-3’

Sequencing primer RAseq: 5’-CTTGCAAAATGGCGTTAC-3’

**Sequencing and identification of retroviral insertion sites**

Based on the gel pattern of the LM-PCR results, some of the bands were rationally selected for sequencing to confirm results from the band patterning on gel electrophoreses.

DNA sequencing was performed by GATC-Biotech AG, Konstanz using an ABI 3730x1 DNA Analyzer. DNA bases, corresponding to the retroviral vector and to the polylinker were removed from the sequences and database search was performed to locate the genomic position of the obtained sequence-fragments. The BLAST (<http://blast.ncbi.nlm.nih.gov>) and the Ensembl (<http://www.ensembl.org>) mouse genome databases were used. The two closest genes at 5’ and 3’ of the RIS and the genes that were hit directly by the retrovirus integration were listed in Table 1 and Supplemental Table 1/A an 1/B.

On average, 7.3±1.8 GFP+ methylcellulose colonies per mouse were analyzed by LM-PCR and thus a total of 80 colonies were subjected to LM-PCR. All together 105 bands were sequenced.
